# Rehabilitation Assessment System for Stroke Patients Based on Fusion-Type Optoelectronic Plethysmography Device and Multi-Modality Fusion Model: Design and Validation

**DOI:** 10.3390/s24092925

**Published:** 2024-05-03

**Authors:** Liangwen Yan, Ze Long, Jie Qian, Jianhua Lin, Sheng Quan Xie, Bo Sheng

**Affiliations:** 1 School of Mechatronic Engineering and Automation, Shanghai University, Shanghai 200444, China; lw_yan@shu.edu.cn (L.Y.);; 2 Second Affiliated Hospital, School of Medicine, Zhejiang University, Hangzhou 310009, China; 3 Department of Rehabilitation Therapy, Yangzhi Affiliated Rehabilitation Hospital of Tongji University, Shanghai 201619, China; 4 School of Electronic and Electrical Engineering, University of Leeds, Leeds LS2 9JT, UK; s.q.xie@leeds.ac.uk

**Keywords:** rehabilitation assessment, stroke, photoplethysmography, multi-modality fusion, MCNN-LSTM-Attention

## Abstract

This study aimed to propose a portable and intelligent rehabilitation evaluation system for digital stroke-patient rehabilitation assessment. Specifically, the study designed and developed a fusion device capable of emitting red, green, and infrared lights simultaneously for photoplethysmography (PPG) acquisition. Leveraging the different penetration depths and tissue reflection characteristics of these light wavelengths, the device can provide richer and more comprehensive physiological information. Furthermore, a Multi-Channel Convolutional Neural Network–Long Short-Term Memory–Attention (MCNN-LSTM-Attention) evaluation model was developed. This model, constructed based on multiple convolutional channels, facilitates the feature extraction and fusion of collected multi-modality data. Additionally, it incorporated an attention mechanism module capable of dynamically adjusting the importance weights of input information, thereby enhancing the accuracy of rehabilitation assessment. To validate the effectiveness of the proposed system, sixteen volunteers were recruited for clinical data collection and validation, comprising eight stroke patients and eight healthy subjects. Experimental results demonstrated the system’s promising performance metrics (accuracy: 0.9125, precision: 0.8980, recall: 0.8970, F1 score: 0.8949, and loss function: 0.1261). This rehabilitation evaluation system holds the potential for stroke diagnosis and identification, laying a solid foundation for wearable-based stroke risk assessment and stroke rehabilitation assistance.

## 1. Introduction

According to a study published in the international journal *The Lancet*, which focused on 369 common diseases, stroke, ischemic heart disease, and diabetes were identified as the three major threats to human health. Among these diseases, China bears the most severe burden of stroke [1]. The China Stroke Prevention and Control Report (2023) states that China has the highest number of stroke patients globally, and the incidence is increasing among younger populations [2]. Stroke is a neurological symptom and disease caused by damage to specific areas of the brain [3,4]. Stroke is considered one of the most severe diseases in modern society as it can lead to death in severe cases and cause physical and mental disabilities. Rehabilitation assessment after stroke has been proven to be beneficial and essential for most stroke patients [5,6]. Julie Bernhardt et al. [7] highlighted the crucial role of rehabilitation in stroke recovery during the First Stroke Recovery and Rehabilitation Roundtable. Rehabilitation assessment not only improves patients’ functional abilities but also enhances their quality of life and social participation.

Commonly used assessment scales include the Brunnstrom Assessment [8], the National Institutes of Health Stroke Scale (NIHSS) [9,10], and the Modified Rankin Scale [11]. The Brunnstrom staging is widely used in clinical practice to classify the rehabilitation progress of stroke patients due to its simplicity and effectiveness. The Brunnstrom staging divides stroke recovery patients into stages of flaccidity, spasticity, voluntary movement, and recovery. By combining changes in neuro-reflex muscle strength and muscle tone at different stages, specific training methods can be applied to achieve the goal of restoring patients’ limb and cognitive functions [12,13,14]. However, most traditional assessment methods are time-consuming, require significant effort, and lack diversity in approaches. Moreover, rehabilitation assessment by healthcare professionals heavily relies on personal experience and subjective judgment, making it difficult to obtain objective and accurate quantitative assessment results that intuitively reflect the patient’s rehabilitation status.

To address these issues, numerous researchers have proposed digital methods for assessing the rehabilitation statuses of patients. Common biomedical sensing technologies include surface electromyography (sEMG), motion analysis systems, electrocardiography, and photoplethysmography (PPG). For example, Bo Sheng et al. [15] utilized the Kinect v2 depth sensor to capture motion data and applied singular spectrum analysis and the multi-ReliefF method to assess upper limb function in stroke hemiparetic patients. Hsin-Ta Li [16] used inertial measurement units (IMUs) and surface electromyography (sEMG) to acquire lower limb motion signals and employed the Support Vector Machine (SVM) algorithm to assess lower limb function in hemiparetic patients. However, the primary goal of stroke rehabilitation is to restore overall functional abilities, and assessing the functions of specific muscles or movements may not comprehensively reflect a patient’s overall motor capabilities. Additionally, in Hsin-Ta Li’s study [16], when participants performed finger movements or movements such as dorsiflexion and knee extension, the signals for each action were manually trimmed through a user interface, introducing subjectivity to the data collection. On the other hand, some researchers have focused on evaluating stroke patients from the perspective of blood signals. For instance, Pei-Wen Huang et al. [17] proposed a multi-modality analysis method incorporating electrocardiography, arterial blood pressure (ABP), and PPG to predict the functional status of stroke patients, achieving an accuracy of 82.7%. However, these studies faced challenges of low identification accuracy and precision.

In recent years, the continuous development of biosensing technology has led to widespread attention to photoplethysmography (PPG) as a convenient and non-invasive monitoring method. The PPG signal waveform reflects changes in blood volume within the measured area. It provides important insights into cardiac ejection capacity and hidden pathological information during blood propagation along the vascular tree [18,19,20]. However, different wavelengths of PPG signals exhibit varying sensitivities to pathological responses, leading to the need for research on multi-sensor PPG systems to obtain comprehensive and accurate physiological information [21]. Previous studies have shown a close correlation between green light PPG signals and electrocardiogram (ECG) R–R intervals [22]. In contrast, red and infrared lights have stronger penetration capabilities, allowing for deeper penetration into deeper layers of vascular tissue [23]. For example, Yuka Maeda et al. revealed the advantages of green-light PPG in pulse rate measurement while the performance of infrared-light PPG in this aspect was relatively poorer [24]. Additionally, Revati Shriram et al. utilized the characteristics of red and infrared lights to investigate the relationship between arterial stiffness and PPG waveforms, providing a foundation for the future assessment of cerebrovascular health [25]. However, the application of PPG signals in the rehabilitation assessment of stroke still faces challenges. In light of this, this study aimed to explore a multi-sensor PPG evaluation system that comprehensively considers red, green, and infrared lights, further investigating the application of multi-wavelength fusion PPG signals in the rehabilitation assessment of stroke.

In previous research, Wei et al. [26] designed a PPG acquisition device and proposed a model that combines the Convolutional Neural Network (CNN), Long Short-Term Memory (LSTM), and an attention mechanism This model achieved a good accuracy rate of 99.1% in the diagnosis and identification of hypertension. However, when using this model for stroke-patient rehabilitation assessment, the accuracy rate was found to be relatively low. To address the aforementioned issues, this paper proposes a new device and system aiming to provide a more convenient data acquisition solution and higher accuracy assessment results. Specifically, the objectives are as follows:

1. To design a fusion-based PPG sampling device named “NeuroPulseGuard” with higher accuracy, safety, reliability, and portability;
2. To propose a multi-modality assessment model (MCNN-LSTM-Attention) based on the fusion of multiple PPG signals. In this study, a total of eight patients and eight healthy individuals were recruited for data collection and clinical experiments. Performance validation and assessment were conducted by comparing the performance of different models on the same dataset, using accuracy rate, precision, recall, F1 score, and computational efficiency as evaluation metrics.

## 2. Materials and Methods

**Figure 1:**
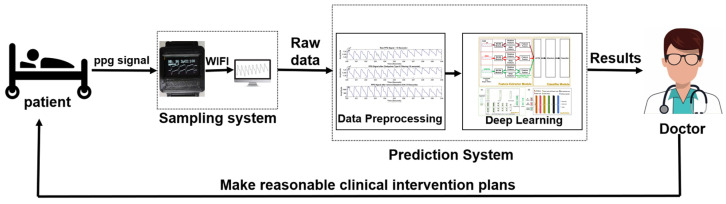
The framework of the stroke assessment system.

Figure 1 illustrates the operational workflow of the system developed in this study. The process consists of the following stages:

1. Designing and implementing a secure, reliable, and portable fusion-based PPG sampling device for collecting PPG data from stroke patients with varying degrees of severity and transmitting the data via Wi-Fi;
2. Preprocessing the data from patients with different severity levels and healthy volunteers;
3. Analyzing the data using the proposed MCNN-LSTM-Attention model to provide rehabilitation assessment grades for stroke patients;
4. Physicians can employ these results for more informed clinical interventions, thereby facilitating better rehabilitation outcomes for the patients.

### 2.1. Equipment

**Figure 2:**
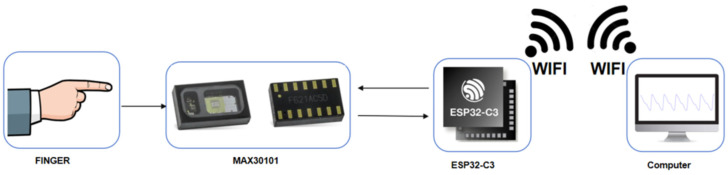
The framework of the PPG acquisition system.

In order to obtain high-quality raw PPG signals, a comprehensive signal acquisition process was designed in this study, as shown in Figure 2. The system consists of the following steps: (1) The PPG photoplethysmography (PPG) sensor module collects PPG signals from the patient’s finger; (2) The PPG sensor interacts with the microcontroller through the I2C protocol; (3) The microcontroller exchanges data with the PC via Wi-Fi.

**Figure 3:**
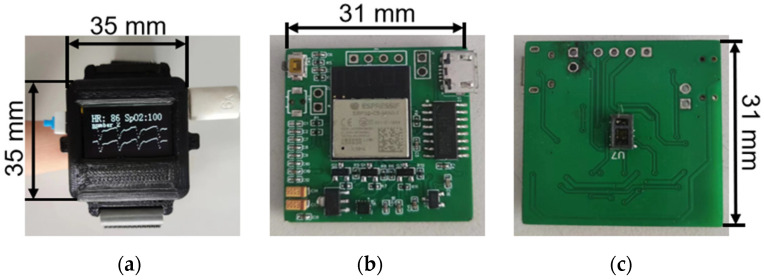
‘NeuroPulseGuard’: (**a**) physical diagram of the fusion-based PPG sampling device; (**b**) front view of the internal PCB board of the fusion-based sampling device; (**c**) schematic diagram of the reverse side of the internal PCB board of the fusion-based sampling device.

**Figure 4:**
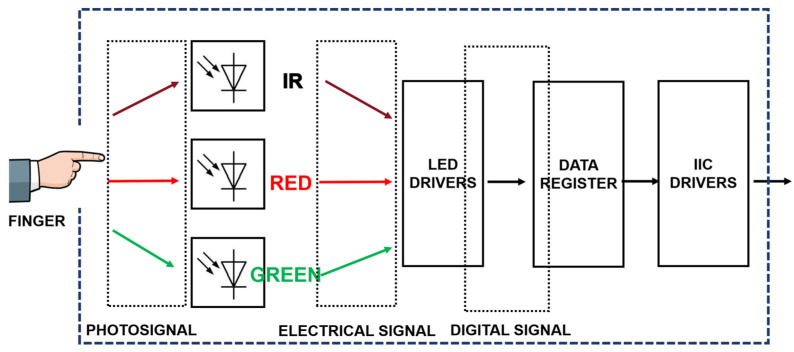
Operational diagram of the multi-functional sensor MAX30101.

As shown in Figure 3, the NeuroPulseGuard device’s primary function is to acquire, transmit, and store PPG signals. The device’s dimensions are 35 mm × 35 mm, with the core PCB board measuring only 31 mm × 31 mm. The chosen microcontroller for this device is the ESP32-C3, an IoT chip known for its secure stability, low power consumption, and cost-effectiveness. It is equipped with a 32-bit single-core RISC-V processor clocked at up to 160 MHz. The chip supports 2.4 GHz Wi-Fi and Bluetooth 5 (LE) and has obtained CLS-Ready certification, complying with device network security standards [27]. Additionally, the device utilizes the MAX30101 as the core component of its multi-sensor system. The MAX30101 is a sensor chip integrating green, infrared, and red light sensors, exhibiting exceptional performance in measuring blood volume changes [28]. As depicted in Figure 4, the MAX30101 design integrates various components such as photodetectors, analog-to-digital converters (ADCs), and digital signal processors (DSPs) to achieve high-precision measurement of blood volume changes. The chip’s LED light sources include green, red, and infrared lights, with peak wavelengths of 527 nm, 660 nm, and 880 nm, respectively, chosen based on their correlation with blood reflection characteristics. The green light wavelength primarily reflects superficial blood volume changes while the red light wavelength is more sensitive to changes in superficial vessels. The infrared light wavelength can penetrate deeper into vascular tissues, providing more comprehensive information [23]. By analyzing signals from these wavelengths comprehensively, a broader and more accurate assessment of blood volume changes can be obtained, enhancing the system’s capability for rehabilitation assessment of stroke.

To validate the functionality and accuracy of the experimental device, a comparative experiment was conducted involving three participants (including two males and one female). The participants’ ages were 45, 52, and 23 years, respectively. During the experiment, the participants were instructed to maintain a supine position on the bed, ensuring a stable physiological state. The PHILIPS DB12 pulse oximeter was used to collect data from the participants’ left index fingers while the experimental device was used to capture PPG signals from their right index fingers. Each data collection sequence lasted for 5 s. The obtained blood oxygen saturation and heart rate data were compared with the records from the PHILIPS DB12 pulse oximeter. To ensure reliability, each participant underwent three measurements. The experimental results are presented in Table 1 and Table 2. In the experimental device, red and infrared light are highly absorbed by blood and can reflect the oxygenation level of hemoglobin. Therefore, red and infrared light were utilized to calculate blood oxygen saturation. On the other hand, green light with a wavelength that can penetrate the skin and superficial blood vessels is highly sensitive to small blood volume changes caused by cardiac pulsations. Consequently, the green light PPG signal was used to calculate heart rate [22]. The heart rate and blood oxygen saturation data, obtained through the acquisition and processing of the PPG signal, were compared with the corresponding data recorded by the PHILIPS DB12 pulse oximeter. The results demonstrated an error of less than 1%, ensuring the accuracy of data acquisition.

### 2.2. Data Processing

The objective of preprocessing filtering is to remove or attenuate noise, artifacts, and interference to extract the desired physiological signals. PPG signals of different wavelengths are, fundamentally, intensity signals collected by optical sensors, sharing similar characteristics and spectral content [29]. Therefore, the same filtering approach was applied for preprocessing in this study.

In the process of PPG signal acquisition, device component characteristics, external environment, and unconscious movements can introduce different frequencies of noise. These noise types can be mainly classified into three categories [30]: random noise, low-frequency noise, and high-frequency noise. Random noise originates from interference by light and other electromagnetic signals in the environment, affecting the intensity of the PPG signal. Low-frequency noise is primarily caused by baseline drift and motion artifacts, including baseline drift due to respiration and short-term tissue deformation caused by motion. High-frequency noise comes from muscle interference and power line interference, appearing as “spike-like” signal noise. Researchers have developed various types of filters to address these issues, such as FIR filters [31,32], Kalman filters [33], Butterworth filters [34,35], moving average filters [36], and Chebyshev Type II filters. According to relevant researchers’ studies [37], by comparing nine different filters with 10 different orders, it was found that the Chebyshev Type II filter exhibited superior performance in improving the quality of the PPG signal. This filter demonstrates frequency selectivity, effectively filtering out interference and noise while preserving valuable information in the signal. Additionally, this study also compared the filtering effects of different filters. Based on the results presented in Table 3, the Chebyshev Type II filter performed the best in terms of signal-to-noise (SNR) ratio.

The Chebyshev Type II filter is a type of digital filter characterized by having minimal ripple within the passband and maximum attenuation within the stopband. This filter allows for the existence of ripples within the passband to achieve a steeper filter response and provides maximum attenuation within the stopband to suppress unwanted frequency components. To describe the transfer function, we use the following formula(s):

(1) $$H(s)=1/sqrt(1+\varepsilon^{2}\times {C_{2}}^{2}(s))$$

Here, H(s) represents the transfer function of the filter, where s is a complex variable. C_2_ denotes the polynomial form of the transfer function for the Chebyshev Type II filter. ε represents the maximum passband ripple parameter. The expression for C_2_(s) is given by the following equation:

(2) $$C_{2}(s)={\left(s^{2}-1\right)}^{2}/(C_{1}(s)\times C_{2}(s)\times \ldots \times C_{n}(s))$$

Here, n represents the order of the filter, and D_1_(s), D_2_(s), …, D_n_(s) are first-order polynomials. They can be expressed using the following formula:

(3) $$D_{k}(s)=s+\alpha_{k}$$

Here, $\alpha_{k}$ represents constants associated with the poles of the filter.

**Figure 5:**
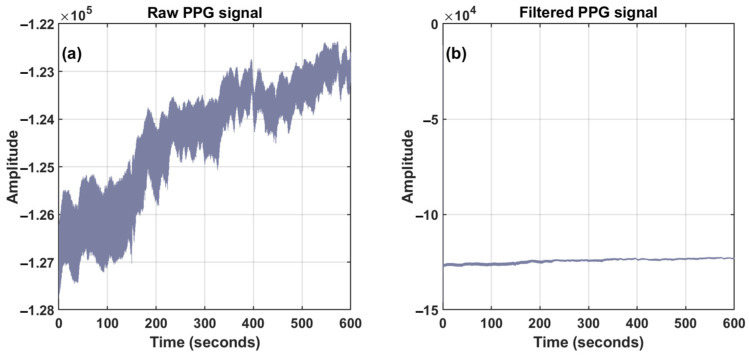
Data processing: (**a**) raw PPG signal (10 min); (**b**) filtered PPG signal (10 min).

**Figure 6:**
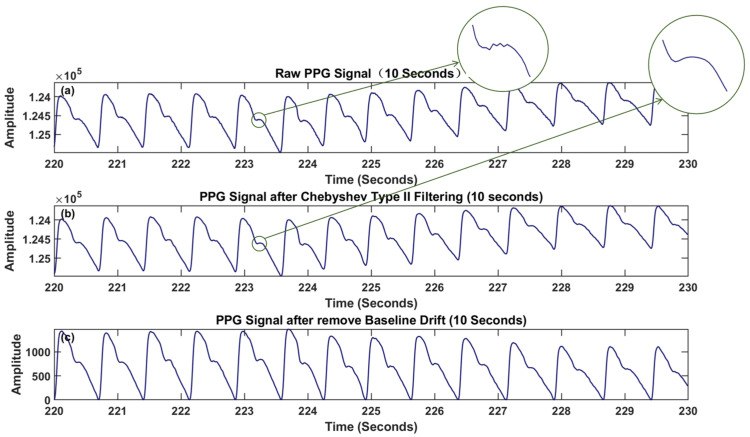
Data processing details: (**a**) raw PPG signal (10 s); (**b**) PPG signal after Chebyshev Type II filtering (10 s); (**c**) PPG signal after removing baseline drift (10 s). The enlarged view of a specific detail in the PPG signal is shown within the green circle.

From Figure 5a,b and the 10-min processed graph, it can be observed that the signal, after undergoing Chebyshev Type II processing, removed a significant portion of baseline drift, and the waveform distribution is more concentrated around the central axis. From Figure 6a,b and the 10-s processed detailed graph, it can be seen that the signal curve, after Chebyshev Type II processing, appeared smoother. From Figure 6b,c and the processed detailed graph, it can be observed that the residual offset component in the signal, after the previous processing, was removed using cubic spline interpolation [38].

### 2.3. Model Architecture

**Figure 7:**
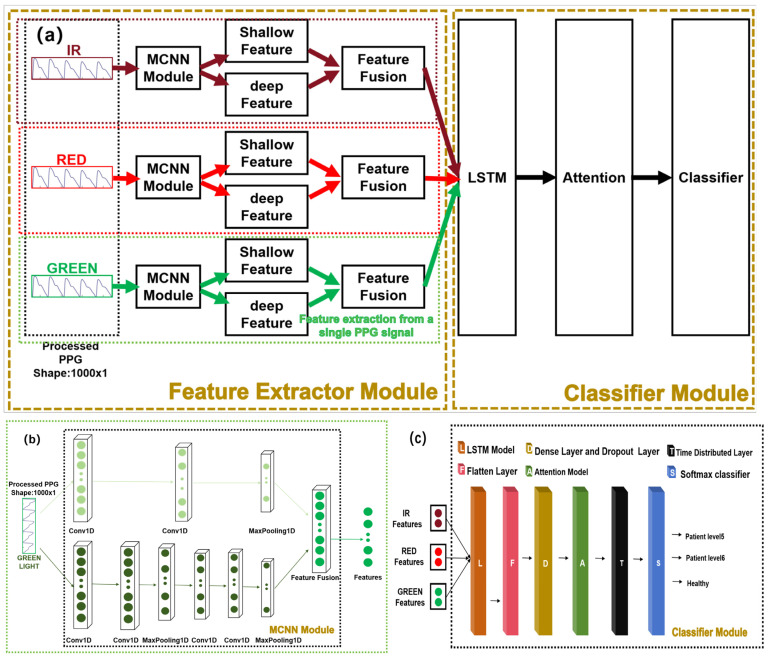
(**a**) MCNN-LSTM-Attention model; (**b**) MCNN module; (**c**) classifier module.

This study aimed to achieve automated classification of stroke patients to provide convenient and accurate rehabilitation assessment. To this end, we have designed an end-to-end deep learning model for extracting features and classifying strokes from preprocessed raw signals. Figure 7a illustrates the overall architecture of the model, consisting of a feature extractor module, depicted in Figure 7b, and a classifier module, shown in Figure 7c. Initially, the preprocessed green, red, and infrared light signals are fed into a Multi-Channel Convolutional Neural Network (MCNN) module, which extracts both shallow and deep features from each signal and subsequently combines them. The fused features from different PPG signals are then input into a Long Short-Term Memory (LSTM) module for further fusion learning. The output of the LSTM module is subsequently passed through an attention module. Finally, the features from the attention module are fed into a classifier for rehabilitation assessment of stroke.

Figure 7b showcases the feature extractor module using the preprocessed green light signal as an example. It comprises two one-dimensional Convolutional Neural Networks (CNNs) with different kernel sizes and depths. These CNNs simultaneously process the preprocessed green light signal as input and extract features in different frequency domains. The larger receptive field is employed for extracting shallow features while the smaller receptive field is utilized for extracting deep features. Using one-dimensional CNNs as feature extractors offers several advantages: firstly, they can automatically learn the underlying meanings of different PPG signals; secondly, weight-sharing strategies significantly reduce the number of parameters in high-dimensional input vectors. The classifier module depicted in Figure 7c consists of hierarchical Long Short-Term Memory (LSTM) networks, an attention layer, and fully connected layers. These components construct a complex nonlinear model that captures the relationship between inputs and outputs. In this architecture, the LSTM module further integrates the features extracted from different PPG signals and serves as an input to the attention layer. The attention mechanism identifies and emphasizes the most important features as determined by the model, thereby enhancing classification performance. The output of the attention layer is then passed to the fully connected layers for classification. Finally, the softmax function is applied to obtain classification results at different levels. Dropout strategies and 10-fold cross-validation were employed to mitigate overfitting during model training.

#### 2.3.1. Single-Signal Fusion Module

**Figure 8:**
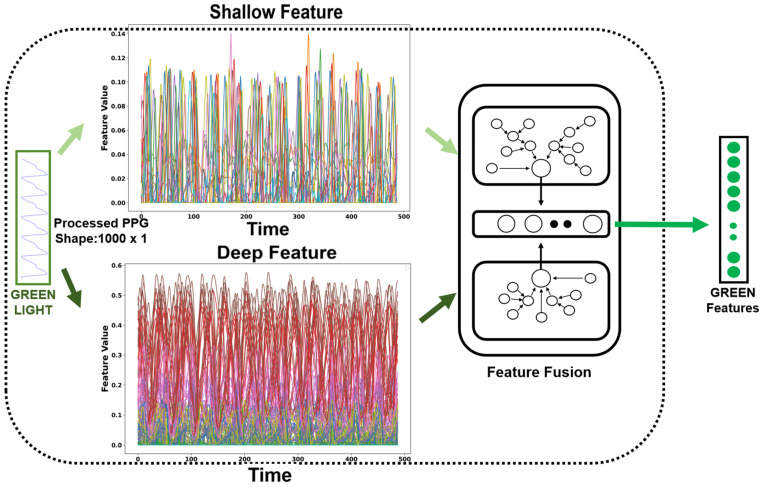
Fusion diagram of MCNN. In the “shallow feature” graph and the “deep feature” graph, different colors represent different features.

Convolutional Neural Networks (CNNs) are feedforward neural networks with convolutional computations and deep structures that are widely used in image processing and natural language processing [39]. They extract features from input data by performing convolutional computations and deep structures, providing effective feature representations. Multi-Channel Convolutional Neural Networks (MCNNs) have been applied in various fields. Liang et al. [40] utilized a multi-channel CNN structure where each channel received a different pre-trained word vector as input. These multiple channels captured additional semantic information from input sentences, enabling the model to learn more discriminative semantic features and have stronger representation capability in natural language processing. X. Chen et al. [41] employed two CNNs with different kernel sizes to extract signal features of different frequencies from raw data and used Long Short-Term Memory (LSTM) networks to classify fault types based on these features. When applying MCNNs to process one-dimensional pulse waveforms (PPG) signals, the following advantages can be obtained:

1. Capturing multi-modal features: PPG signals are measurements of changes in blood volume caused by heartbeats obtained through optical sensors. PPG signals contain components with different frequencies and amplitudes that are related to physiological parameters such as heart rate and blood pressure. By using multiple channels in convolutional layers, MCNN can capture features at different scales simultaneously. For example, lower-frequency channels can capture the overall shape and fluctuations of heartbeats while higher-frequency channels can capture subtle variations in heartbeats [42,43,44].
2. Multi-modal fusion: PPG signals can be obtained from three different light sources, each providing slightly different characteristics in PPG signals. MCNN can process PPG signals from different lights simultaneously and extract feature representations for light sources through multi-channel convolutional layers. By applying multi-channel convolution and pooling operations, MCNN can fuse the information from different light sources into a unified feature representation, enhancing the model’s understanding of PPG signals.
3. Hierarchical feature extraction: Hierarchical feature extraction is an important characteristic of MCNN, typically comprising multiple convolutional and pooling layers. This hierarchical structure enables the progressive extraction of features at different levels of abstraction from PPG signals. As shown in Figure 8, taking the green light PPG signal as an example, MCNN utilizes CNNs to extract deep and shallow features separately. These features originate from different layers and exhibit distinct characteristics. The shallow convolutional layers aim to capture low-level features of PPG signals, such as the shape and fluctuations of heartbeats, to preserve the local information of the signals better. As the network layers deepen, the deep feature encoder can capture more abstract and complex patterns in the signal, such as patterns of heart rate variations or cardiac pathologies [45,46]. By combining these two types of features from different levels, MCNN can fully leverage both global and local information in the signal, resulting in more comprehensive and accurate feature representations. The fused features are then input into the Long Short-Term Memory (LSTM) network for further temporal modeling and processing. Consequently, hierarchical feature extraction enables MCNN to represent and classify PPG signals better, thereby improving the model’s performance and robustness.

One-dimensional convolution is used for feature extraction for one-dimensional time series data such as PPG signals.

(4) $$y_{i}^{k+1}\left(j\right)=w_{i}^{k}\times X^{k}\left(j\right)+b_{i}^{k}$$

Here, w and b are the weights and biases of the i-th layer filter and the k-th layer, respectively, and X represents the j-th local input in the k-th layer.

The i-th layer of the k + 1 channel after pooling can be described as follows:

(5) $$P_{i}^{k+1}\left(j\right)={max}_{\left(j-1\right)W+1\leq t\leq jW}\{q_{i}^{k}(t)\}$$

q represents the t-th neuron in the i-th channel of the k-th layer and W is the width of the pooling kernel.

In PPG signal processing, one-dimensional convolutional neural networks can be used to extract temporal features from the signals. The convolutional layer learns filters suitable for PPG signals to capture important features while the pooling layer further reduces the dimensionality of the features and retains the main signal patterns. These features can be utilized in various applications such as heart rate detection, emotion analysis, and disease diagnosis.

#### 2.3.2. Multi-Signal Fusion Module

Pulse wave data belong to the category of one-dimensional temporal data, and the recurrent neural network (RNN) structure in deep learning algorithms has shown promising results in processing time series data [47]. However, RNNs are prone to gradient vanishing or exploding problems. Therefore, in this paper, the Long Short-Term Memory (LSTM) network structure is employed as a substitute for the RNN model.

**Figure 9:**
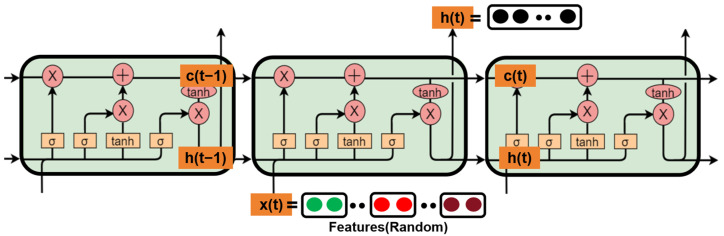
Diagram of LSTM multi-signal fusion structure.

As shown in Figure 9, the LSTM consists of three gating mechanisms: the forget gate, the input gate, and the output gate. The PPG signal features extracted by the Multi-Channel Convolutional Neural Network (MCNN) are fed into the input gate. The forget gate determines the amount of information to retain in the previous time step’s state. It computes the activation value of the forget gate by considering the current input, the previous output, and the state information, followed by applying the sigmoid function. The activation value of the forget gate is multiplied by the state of the previous time step, controlling which information should be forgotten from the previous state.

The specific formulas are as follows; the information received by the forget gate includes the current input $x_{t}$, the previous node’s output $h_{t-1}$, and the previous node’s state $C_{t-1}$. The activation calculation formula for the forget gate (where b and W are bias and weight vectors, and σ denotes the sigmoid function) is presented below:

(6) $$f_{t}=\sigma \left(W_{f}\times \left[x_{t},h_{t-1},C_{t-1}\right]+b_{f}\right)*C_{t-1}$$

(7) $$i_{t}=\sigma \left(W_{i}\times \left[x_{t},h_{t-1},C_{t-1}\right]+b_{i}\right)$$

(8) $$C_{t}=f_{t}+i_{t}\times \tanh\left(W_{c}\times \left[x_{t},h_{t-1},C_{t-1}\right]+b_{c}\right)$$

Based on the new state $C_{t}$ of this neuron node, the LSTM structure can output the current neuron’s output by using the output gate based on $h_{t-1}$, $x_{t}$, and the new state $C_{t}$.

(9) $$o_{t}=\sigma \left(W_{o}\times \left[x_{t},h_{t-1},C_{t}\right]+b_{o}\right)$$

(10) $$h_{t}=\tanh\left(C_{t}\right)\times o_{t}$$

#### 2.3.3. Accuracy Improvement Module

**Figure 10:**
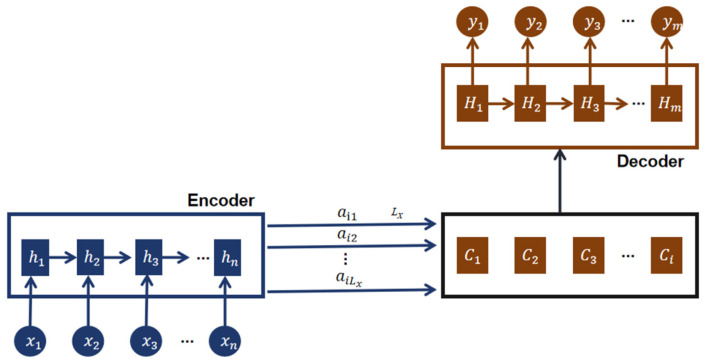
Abstract Encoder–Decoder framework.

The attention mechanism simulates the behavior of human vision or attention, allowing the model to focus more on important parts of the input sequence [48,49]. General attention mechanisms are based on the Encoder–Decoder framework, as shown in Figure 10. The purpose of this model is to address the task of mapping variable-length input sequences $X=(x_{1},x_{2},\ldots ,x_{n})$ to variable-length output sequences $Y=(y_{1},y_{2},\ldots ,y_{m})$. The Encoder is responsible for receiving the input sequence X and transforming it into an intermediate abstract representation C through nonlinear transformations: $C=f(x_{1},x_{2},\ldots ,x_{n})$. The primary role of the Encoder is to encode the input sequence into a fixed-dimensional representation, capturing the semantic and contextual information of the input sequence. The Decoder’s task is to predict and generate the output at time step i, $y_{i}$, based on the intermediate abstract representation C of the input sequence X and the previously generated partial outputs $y_{1},y_{2},\ldots ,y_{i-1}:y_{i}=g(y_{1},y_{2},\ldots ,y_{i-1},C)$. The Decoder utilizes the intermediate representation C and the already generated partial outputs to infer the next output, gradually constructing the output sequence.

$H_{m-1}$ represents the hidden state of the Decoder at time step m−1, $y_{m}$ is the target value, and $C_{i}$ is the context vector. Therefore, the hidden state at time step m can be defined as follows.

(11) $$y_{m}=f\left(H_{m-1},y_{m-1},C_{i}\right)$$

$C_{i}$ depends on the hidden vectors of the input sequence on the Encoder side and can be represented after weighted processing, as shown in Equation (12).

(12) $$C_{i}=\sum_{j=1}^{L_{x}}\alpha_{ij}h_{j}$$

$h_{j}$ represents the hidden vector of the j-th value on the Encoder side, which contains information from the entire input sequence but focuses on the surrounding portion of the j-th value. $L_{x}$ is the length of the input side, $\alpha_{ij}$ represents the attention allocation coefficient of the j-th value on the Encoder side to the i-th value on the Decoder side, and the sum of $\alpha_{ij}$ probabilities is 1. The calculation formula for $\alpha_{ij}$ is shown in Equation (13).

(13) $$\alpha_{ij}=\frac{exp(\alpha_{ij})}{\sum_{j=1}^{L_{x}}exp(\alpha_{ij})}$$

(14) $$\alpha_{ij}=a\left(y_{m-1},h_{j}\right)$$

$\alpha_{ij}$ represents an alignment model that measures the alignment degree between the value at position j on the Encoder side and the value at position i on the Decoder side. Introducing an attention mechanism in the model can help capture the temporal correlation in the signal better and enable the model to adapt to the signal characteristics of each patient individually, improving the personalization effect of classification.

In summary, this model uses MCNN for feature extraction to learn signal features in different frequency domains automatically. Then, LSTM–Attention networks are used for temporal modeling and internal feature extraction, followed by fully connected layers for classification. This structure effectively handles PPG signals and converts them into classification results for different levels of stroke patients.

## 3. Experiments

### 3.1. Participants

The subjects were divided into two groups: the stroke patient group and the healthy group. The stroke patient group consisted of 8 individuals diagnosed with ischemic stroke at the Sunshine Rehabilitation Center in Shanghai, China. Correspondingly, during the same period, 8 male volunteers of similar age were selected as the healthy group, with an age range of 50 ± 10 years (Table 4). The average age of the healthy control group was 54.125 years, with a standard deviation of 4.0510, while the average age of the stroke patient group was 49.875 years, with a standard deviation of 8.9512. Previous studies have shown that age is a key factor influencing pulse wave characteristics [50], and many researchers are exploring methods for assessing vascular aging [51,52]. Parameters such as pulse transit time [53] and pulse wave velocity [54] are considered standards for evaluating vascular stiffness. Some researchers have noted a significant correlation between PPG features obtained from the finger and age [55]. This feature resembles the age-related progression of arterial stiffness assessed through pulse wave velocity (PWV). Therefore, age is a factor that influences the evaluation of the model. It is worth noting that both groups included only male participants, ensuring gender consistency in the study.

The Brunnstrom Assessment Scale is a grading system used to describe the ex-tent of limb movement recovery in patients. This system consists of six stages, numbered from 1 to 6, representing different levels of limb movement recovery. However, in this paper, the seventh stage is defined as representing normal activity exhibited by individuals who have fully recovered and are in a healthy state. The specific stages are illustrated in Table 5. The stroke patients all experienced their first ischemic stroke within three months. Among these individuals, 6 patients exhibited right-sided hemiplegia and 2 patients exhibited left-sided hemiplegia. Of these individuals, 4 patients were classified as being in stage V and 4 patients as being in stage VI. The selection of participants in stages V and VI was primarily based on the feasibility of data collection and the practical applicability of the study. Firstly, patients in stage I are in the acute phase, during which their neurological and physiological states undergo significant changes and adjustments [56,57,58,59]. Therefore, collecting data during this stage may cause secondary harm to the patients. Secondly, patients in stages II, III, and IV typically exhibit more pronounced spasms [12,14], and the significant fluctuations caused by spasms introduce excessive noise, making the collected data less accurate and reliable. Please note that the study design was intended for individuals aged 40 to 60, in stages V and VI, and for healthy individuals. Participants outside this range may not be applicable to this model.

**Figure 11:**
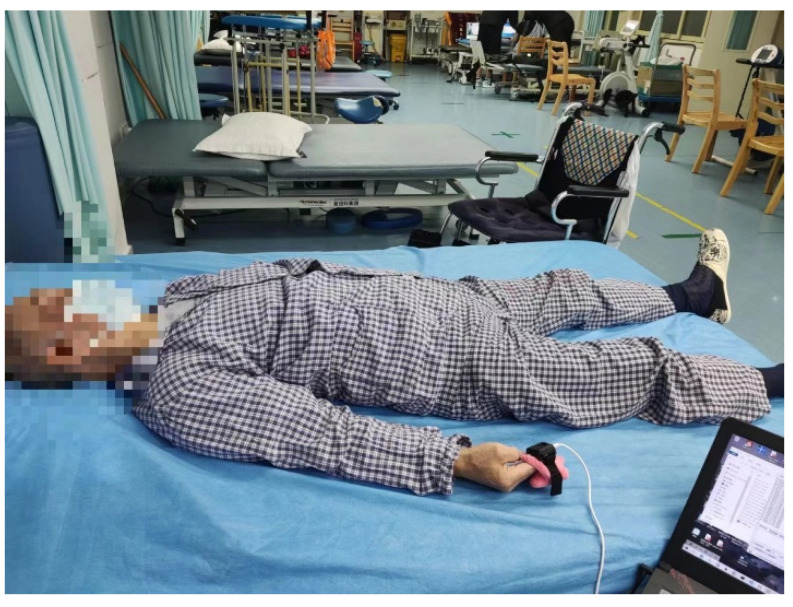
Photo of PPG signal acquisition of a patient in the hospital.

The basic information on all volunteers, including age, gender, type of hemiplegia, and alcohol history, was collected. Simultaneously, the patients’ PPG signals were recorded. The control room temperature was maintained at 23 °C while the finger temperature was set at 32 °C. Participants were instructed to remain in a stationary state. The PPG signals from the right index finger of each volunteer were collected continuously for 30 min during the sampling period, which took place between 17:00 and 19:00 in the afternoon. The sampling frequency was set at 100 Hz. The data collection experiment is depicted in Figure 11.

### 3.2. Testing Protocol

The engagement of stroke patients in this study’s voluntary activities underwent a stringent ethical review process, adhering to the principles outlined in the Helsinki Declaration [60]. The primary objective was to safeguard the participants’ rights and ensure ethical compliance throughout the activities. A comprehensive set of measures was implemented to protect the participants’ welfare. Firstly, prior to commencing the experiments, detailed procedural instructions were provided to each participating subject, accompanied by comprehensive disclosure regarding the data collection requirements and the research objectives, processes, and objectives for which the data would be utilized. Secondly, this study placed utmost importance on preserving the privacy and confidentiality of the participants, ensuring the proper handling and protection of their personal information and medical records. The volunteers were given the autonomy to participate in the activities willingly and possessed the right to withdraw at any point without facing any detrimental repercussions. Stringent adherence to ethical principles was maintained throughout the study, ensuring that the activities were conducted in alignment with the highest moral standards while safeguarding the interests and safety of the participants to the greatest extent possible. The volunteers provided informed consent and signed consent forms while the research protocol underwent thorough deliberation and approval by the Ethics Committee of Shanghai University.

### 3.3. Validation

#### 3.3.1. K-Fold Cross-Validation

**Figure 12:**
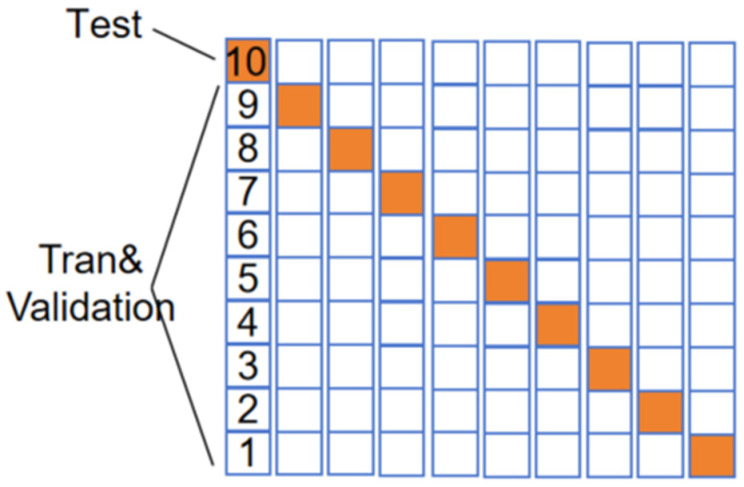
Ten-fold cross-validation.

As shown in Figure 12, K-fold cross-validation is a commonly used technique in machine learning to evaluate and validate model performance [61]. Firstly, the original dataset is divided into 10 equally sized subsets, and then, 10 iterations are performed. In each iteration, one of the subsets is selected as the validation set while the remaining 9 subsets are used as the training set. Each subset is used as the validation set once, allowing for multiple rounds of training and validation, which provides a reliable estimate of the model’s performance. Ten-fold cross-validation helps detect whether a model is overfitting the training data. If the model performs well on each validation set, it indicates a strong generalization capability.

#### 3.3.2. Performance Evaluation

The model’s performance was comprehensively evaluated by calculating the probability distribution of the dataset being stroke-related and the overall accuracy, precision, recall, and F1 score.

The accuracy was computed using Formula (15):

(15) $$Accuracy=\frac{TP+TN}{TP+TN+FP+FN}$$

The precision was calculated as shown in Formula (16):

(16) $$Precision=\frac{TP}{TP+FP}$$

The recall was calculated as shown in Formula (17):

(17) $$Recall=\frac{TP}{TP+FN}$$

The F1 score was calculated as shown in Formula (18):

(18) $$F1=\frac{2\times Precision\times Recall}{Precision+Recall}$$

The relationships between TP, TN, FP, and FN are summarized in Table 6.

## 4. Results

**Figure 13:**
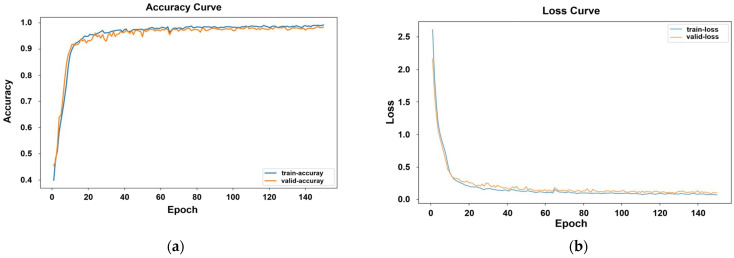
(**a**) Training: accuracy curve. (**b**) Training: loss curve.

The training process is illustrated in Figure 13, showing the accuracy and loss curves during a training iteration. The figures indicate that as the number of iterations increases, the model’s accuracy gradually improves and reaches its peak while the loss value gradually decreases and eventually stabilizes. The validation accuracy is slightly lower than the training accuracy.

**Figure 14:**
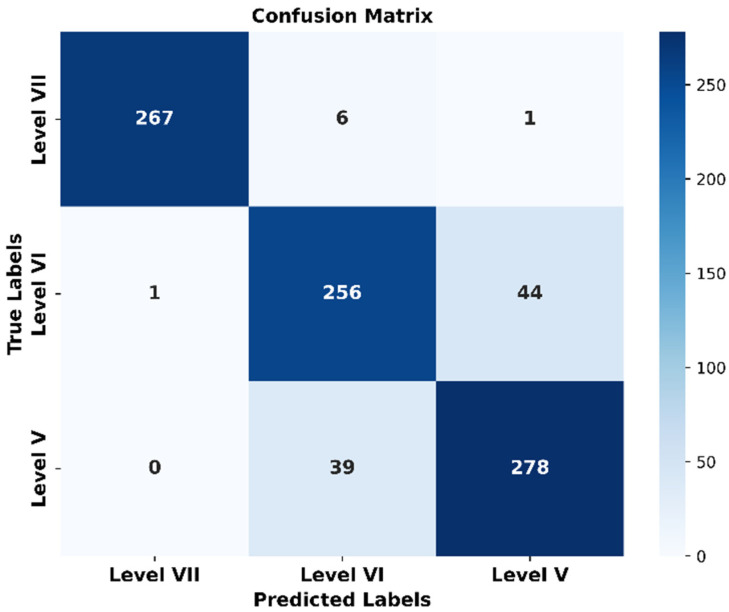
Confusion matrix.

Based on the confusion matrix obtained from the test set (as shown in Figure 14), the model’s recognition accuracy can be calculated as 91.25%, precision as 89.80%, recall as 89.70%, and F1 score as 89.49%. It is important to note that in Figure 14, “Level VII” represents healthy individuals.

The classification results of different models on the same dataset are presented in Table 7, representing the average values from 10-fold cross-validation. The MCNN-LSTM-Attention model achieves the highest accuracy of 91.25% while the CNN–LSTM model has the lowest accuracy at only 69.04%. The MCNN-LSTM-Attention model outperforms the CNN–LSTM–Attention model by 25.4% in terms of accuracy, and the MCNN–LSTM model surpasses the CNN–LSTM model by 24.8%. The MCNN-LSTM-Attention model achieves a 10.5% higher accuracy than the MCNN–LSTM model, and the CNN–LSTM–Attention model achieves a 5.1% higher accuracy than the CNN–LSTM model.

Table 8 presents the training times required for different algorithm models in a single training iteration. It is worth noting that models with attention mechanisms significantly increase the training time compared to those without attention mechanisms. The difference in training time between the multi-scale CNN model and the single-scale CNN model is minimal.

## 5. Discussion

The main findings of this study were as follows: (1) The fusion-based PPG signal acquisition system designed and investigated using ESP32C3 meets the experimental requirements and exhibits advantages such as portability, low cost, and good accuracy. (2) The proposed multi-modality approach significantly improves the accuracy compared to the single-modal approach, with the CNN–LSTM–Attention stroke recognition model achieving an accuracy of 91.94%. Compared to feature extraction from a single modality, the proposed model better captures the interdependencies among types of PPG sequence information, enhancing the feature representation capability and resulting in performance improvement.

### 5.1. Fusion-Type Device

This paper presents the design of a precision Pulse Plethysmography (PPG) signal acquisition device. The device utilizes an ESP32-C3 microcontroller as its core, offering several advantages over traditional approaches. Firstly, the ESP32-C3 microcontroller enables the collection of PPG signals from the fusion sensor MAX30101, including green, red, and infrared light, facilitating comprehensive signal acquisition for more accurate data in the rehabilitation assessment of stroke. Secondly, the device ensures stable data transmission, high accuracy, low power consumption, and a compact form factor, measuring only 31 mm × 31 mm. It also has a relatively low cost, making it portable and cost-effective.

**Figure 15:**
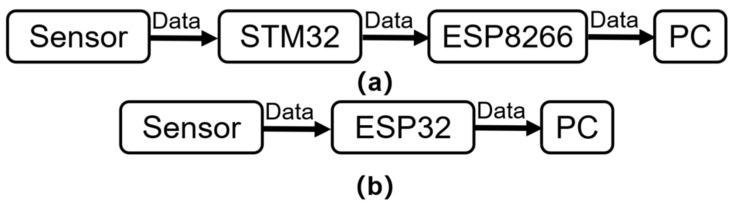
Comparison of the internal structures of the devices: (**a**) Wei’s equipment; (**b**) ‘NeuroPulseGuard’ device.

As shown in Figure 15, in comparison to Wei’s previously designed device, which required multi-level data transmission through sensors, STM32, ESP8266, and Wi-Fi to the PC, the ‘NeuroPulseGuard’ microcontroller ESP32-C3 directly acquires data from the sensor and transmits it via Wi-Fi, simplifying the system architecture and improving system stability and robustness. The device has a smaller form factor, lower power consumption, and lower cost. Table 1 demonstrates that the ‘NeuroPulseGuard’ device, after collecting and processing PPG signals, achieves a heart rate measurement with an error within 1% compared to the heart rate data recorded by the PHILIPS DB12 pulse oximeter, further enhancing accuracy compared to Wei’s device with 2% precision.

Additionally, the ‘NeuroPulseGuard’ device, when compared to those developed by other researchers such as the wrist-worn PPG signal monitoring devices designed by Zhang et al. [62] and Lee et al. [63], offers advantages. Despite sharing the same portability, the ‘NeuroPulseGuard’ device does not suffer from the weaker PPG signal strength that can result from the small and shallow blood vessels at the wrist. Compared to PPG signals obtained from the fingertip, wrist-worn devices may result in higher levels of signal noise. These issues limit their suitability and performance in real-world applications.

### 5.2. Multi-Modality Approach

The proposed multi-modality approach in this paper primarily included feature extraction fusion for a single PPG signal and feature fusion for multiple PPG signals. Specifically, the following statements can be made: (1) The feature extraction fusion for a single PPG signal utilized a MCNN, which introduced multiple convolutional channels compared to a traditional single-channel CNN. This design allowed the MCNN to comprehensively capture features at different levels (deep and shallow), thereby enhancing the model’s feature extraction capability. By utilizing PPG signals from green, red, and infrared light as inputs, the MCNN could extract comprehensive physiological data, providing richer feature information for the rehabilitation assessment of stroke. (2) In terms of feature fusion for multiple PPG signals, previous research has indicated that LSTM exhibited higher accuracy in the field of multi-signal fusion [64,65]. In this study, by integrating PPG signal features from green, red, and infrared light, a temporal dependency was established. This temporal dependency effectively captured the correlation between features and further improved the model’s performance. Additionally, an attention mechanism was introduced to enhance the model’s performance. The attention mechanism dynamically adjusted weights based on the importance of input data, enabling the model to focus more on crucial parts and improve its perception of key features [66].

From the perspective of overall model performance, the proposed MCNN-LSTM-Attention model achieved an accuracy of 91.25% in rehabilitation assessment, surpassing the accuracy of 72.73% for the CNN–LSTM–Attention model and 86.79% for the MCNN–LSTM model. Furthermore, compared to other models, the MCNN-LSTM-Attention model demonstrated good precision (0.8980), recall (0.8910), F1 score (0.8949), and loss function (0.1261) values. These findings indicated that the model exhibited good accuracy and reliability in the rehabilitation assessment of stroke. To fully consider the utilized dataset in the experiments, 10-fold cross-validation was employed to effectively reduce biases arising from uneven dataset partitioning; improve the reliability, stability, and generalization ability of model evaluation; and ensure consistent performance across different data subsets, thus enhancing the credibility of the experimental results. In terms of overall model efficiency, the MCNN-LSTM-Attention model required 7.6 s per training cycle. In comparison, the CNN–LSTM–Attention model required 7.3 s, the MCNN–LSTM model required 1.6 s, and the CNN–LSTM model required 1.3 s. The introduction of the MCNN feature extraction module only increased the training time by 0.2–0.3 s but achieved performance improvement.

In the field of pulse wave analysis using photoplethysmography (PPG), traditional machine learning has been a significant topic. However, previous research has shown that deep learning networks outperform traditional machine learning algorithms in PPG signal processing tasks [18,67,68]. Therefore, in this paper, the deep learning algorithm for PPG patient data is not compared with traditional machine learning algorithms. During the feature extraction process of machine learning, researchers found that the waveform in stroke patients is influenced by vascular wall elasticity, making it difficult to locate the dicrotic wave and even resulting in the disappearance of the dicrotic wave. This leads to missing feature points and limited dimensions of manually extracted features, making it challenging to discover deep-level features and correlations between features.

In comparison with other studies in various multi-signal research fields, this study compared the proposed method with other research results, as shown in Table 9. These studies all involved the rehabilitation assessment of stroke. In the first study [26], Wei et al.‘s model (CNN–LSTM–Attention) achieved only 72.73% accuracy. In the second study [17], based on the NIHSS scale, 234 features were extracted from EKG, ABP, and PPG signals, and a linear kernel SVM classifier was employed, resulting in an accuracy of 82.7%. Furthermore, in the third study by Zhe Zhang et al. [69], patients’ levels were evaluated based on the Brunnstrom scale using a fuzzy inference system, achieving an accuracy of 87.5%. In the fourth study, the C4.5 decision tree algorithm was used for patient-level recognition based on the HINSS scale [70], and an accuracy of 91.11% was achieved. The classification accuracies in these four studies were lower than those of the proposed model in this paper. In comparison, the MCNN-LSTM-Attention rehabilitation assessment of the stroke model proposed in this study can effectively extract deep-level features from PPG signals and improve accuracy through multi-modality feature extraction. In the fifth study [16], based on the Brunnstrom scale, the lower limb movement signals of patients were obtained through inertial measurement units (IMUs) and surface electromyography (sEMG). A total of 480 features were extracted, and an SVM classifier was utilized, resulting in an accuracy of 95.2%. However, in the assessment process, the signals for each movement were manually trimmed from a user interface. The experimental process was complex and cumbersome, and subjective factors were involved, limiting its applicability.

Furthermore, as observed in Table 9, traditional machine learning algorithms seemed to outperform deep learning algorithms in the rehabilitation assessment of stroke. However, the relevant literature suggests that deep learning networks outperform traditional machine learning algorithms in many cases. The reason for this apparent contradiction is that traditional machine learning algorithms make use of multiple signal sources, such as combinations of IMU-ECG, IMU-ECG, EKG-ABP-PPG, and others. These combinations of different signal types provide richer feature information, resulting in improved classification accuracy. Therefore, the high accuracy achieved by traditional machine learning methods is closely tied to the comprehensive utilization of multi-signal data. In contrast, this study focused on the classification of multi-modal PPG signals for the rehabilitation assessment of stroke. The MCNN-LSTM-Attention rehabilitation assessment of the stroke model proposed in this study achieved high accuracy despite using only multi-modal PPG signals and not combining multiple signal sources. This suggests that deep learning methods have an advantage in handling multi-modal data from a single signal source. If multiple signal sources are combined in the future, it is likely that further improvements in accuracy can be achieved. This indicates the potential of the proposed deep learning method in the future.

### 5.3. Limitations and Future Work

The study had certain limitations that could impact the results. Firstly, similar to many studies, the number of volunteers in this experiment was limited. However, the proposed MCNN-LSTM-Attention model focused on the raw data rather than the traditional feature-based algorithms that require feature extraction from raw data. In this preliminary study, a total of 8,640,000 data points (three wavelengths × 100 Hz × 60 s × 30 min × 16 participants) were collected, and 91.25% accuracy was achieved. Secondly, age is a crucial factor influencing PPG features, and this study only targeted participants aged 40 to 60. The model might not be applicable to individuals outside this age range. Lastly, this study only involved stroke patients in stages V and VI, as well as healthy individuals, limiting the generalizability of the model to all stroke patients undergoing rehabilitation.

In the future, the further validation and improvement of the model, as well as the enhancement of its accuracy, generalization, and robustness, can be achieved by collecting more patient data and increasing the sample size. Additionally, seeking higher-quality sensors to enhance hardware and continuously optimizing the deep learning model used in this study will broaden its applicability and scope. Finally, the NeuroPulseGuard data acquisition device, which enables the convenient collection of multi-modal PPG signals, holds the potential to assist post-recovery stroke patients and is suitable for community-wide adoption.

## 6. Conclusions

This paper presents the design and investigation of a fusion-based photoplethysmography (PPG) and capacitive volume pulse acquisition device for the rehabilitation assessment of stroke. Subsequently, a recognition model based on MCNN-LSTM-Attention has been proposed. The incorporation of a multi-modality fusion mechanism and attention mechanism enhances the model’s capability to represent features, leading to improved recognition accuracy. Experimental results demonstrate that the MCNN-LSTM-Attention rehabilitation assessment of the stroke model achieves an average accuracy of 91.9% in 10-fold cross-validation. The proposed model outperforms other deep learning models in terms of precision, accuracy, and F1 score metrics.

## Figures and Tables

**Table 1:** Comparison of device blood oxygen values with the commercial instrument.

No. of Volunteer	Experimental Data/%	DB12/%	Error
1	98	98	0%
	98	97	1%
	96	97	1%
2	95	95	0%
	99	100	1%
	97	97	0%
3	95	96	1%
	97	97	0%
	100	100	0%

**Table 2:** Comparison of device heart rate values with commercial instruments.

No. of Volunteer	Experimental Data/bpm	DB12/bpm	Error
1	86	86	0%
	92	93	1%
	89	89	0%
2	89	90	1%
	99	100	1%
	101	100	1%
3	109	108	1%
	101	101	0%
	86	85	1%

**Table 3:** Comparison of filtering effects among different filters.

	Chebyshev Type II Filter	Gaussian Filter	Savitzky–Golay Filter	Smooth Filter
SNR (dB)	81.9626	40.845	76.5083	45.2653

**Table 4:** Comparison of basic information of volunteers in group 2.

Group	Gender (Male %)	Age (Years)	Mean Age (Years)	Standard Deviation of Age (Years)	Hypertension (%)	Hemiplegia/ Right (%)	Right- Handedness (%)
Healthy	8 (100)	40-60	54.125	4.0510	0	-	8(100)
Patient	8 (100)	40-60	49.875	8.9512	62.5	75	8(100)

**Table 5:** Brunnstrom assessment scale [8].

Stage	Description
Ⅰ Flaccid Stage	No movement was initiated or elicited.
Ⅱ Spasticity Appears	Synergies or components are first appearing. Spasticity is developing.
Ⅲ Increased Spasticity	Synergies or components are initiated voluntarily. Spasticity is marked.
Ⅳ Decreased Spasticity	Movements are deviating from basic synergies. Spasticity is decreasing
Ⅴ Complex Movement Combinations	There is relative independence of basic synergies. Spasticity is waning
Ⅵ Spasticity Disappears	Movement coordination is near-normal. Spasticity is minimal.

**Table 6:** Correspondence of TP, TN, FP, and FN.

Confusion Matrix		Ground Truth	
		Positive	Negative
Predicted value	**Positive**	TP	FP
	**Negative**	FN	TN

**Table 7:** Classification results of different models on the dataset.

Method	Accuracy	Precision	Recall	F1 Score	Loss
CNN–LSTM	0.6904	0.6560	0.6508	0.6534	0.5128
CNN–LSTM–Attention	0.7273	0.6946	0.6362	0.6928	0.4907
MCNN–LSTM	0.8679	0.8861	0.8419	0.8634	0.3677
MCNN-LSTM-Attention	0.9125	0.8980	0.8970	0.8949	0.1261

**Table 8:** Training times for different models (one epoch).

Model	Time/s
CNN–LSTM–Attention	7.3
CNN–LSTM	1.3
MCNN–LSTM	1.5
MCNN-LSTM-Attention	7.6

**Table 9:** Classification performance comparison.

Method	Feature Numbers	Stroke Scale	Classifier	Accuracy
PPG	-	Brunnstrom	MCNN-LSTM-Attention	91.25%
PPG [26]	-	Brunnstrom	CNN–LSTM–Attention	72.73%
EKG-ABP-PPG [17]	234 features	NIHSS	Linear kernel SVM	82.7%
Motion data samples [69]	27 features	Brunnstrom	Fuzzy inference system	87.5%
HINSS [70]	13 features	HINSS	C4.5 decision trees	91.11%
IMU and ECG feature [16]	480 features	Brunnstrom	SVM	95.2%

## Data Availability

Data are unavailable due to privacy and ethical restrictions.
